# Extracts of Polypore Mushroom Mycelia Reduce Viruses in Honey Bees

**DOI:** 10.1038/s41598-018-32194-8

**Published:** 2018-10-04

**Authors:** Paul E. Stamets, Nicholas L. Naeger, Jay D. Evans, Jennifer O. Han, Brandon K. Hopkins, Dawn Lopez, Henry M. Moershel, Regan Nally, David Sumerlin, Alex W. Taylor, Lori M. Carris, Walter S. Sheppard

**Affiliations:** 1 Fungi Perfecti, LLC. Olympia, Washington, USA; 2 Washington State University. Pullman, Washington, USA; 3 USDA-ARS Beltsville, Maryland, USA

## Abstract

Waves of highly infectious viruses sweeping through global honey bee populations have contributed to recent declines in honey bee health. Bees have been observed foraging on mushroom mycelium, suggesting that they may be deriving medicinal or nutritional value from fungi. Fungi are known to produce a wide array of chemicals with antimicrobial activity, including compounds active against bacteria, other fungi, or viruses. We tested extracts from the mycelium of multiple polypore fungal species known to have antiviral properties. Extracts from amadou (*Fomes*) and reishi (*Ganoderma*) fungi reduced the levels of honey bee deformed wing virus (DWV) and Lake Sinai virus (LSV) in a dose-dependent manner. In field trials, colonies fed *Ganoderma resinaceum* extract exhibited a 79-fold reduction in DWV and a 45,000-fold reduction in LSV compared to control colonies. These findings indicate honey bees may gain health benefits from fungi and their antimicrobial compounds.

## Introduction

The Western honey bee (*Apis mellifera*) is a critical component of crop production and food biosecurity worldwide. *A. mellifera* and other members of the genus *Apis* also play a key role in the ecological stability of wild plant communities within areas of endemism in Europe, Africa and Asia. Managed *A. mellifera* colonies are estimated to contribute over $15 billion annually to the US agricultural economy through the pollination of numerous fruits, nuts and vegetables^1^. The pollination of almonds in California alone requires relocating over 75% of the managed honey bee colonies (nearly 2 million) in the United States on this single crop during bloom. Over the past decade, beekeepers have experienced a dramatic increase in annual colony losses, typically averaging well over 30%^2–4^. This combination of high demand and reduced supply has led to expansive increases in pollination costs for growers, while beekeepers have been hard-pressed to maintain adequate numbers of healthy honey bee colonies to remain economically viable, even with the benefit of higher pollination service fees.

**Figure 1:**
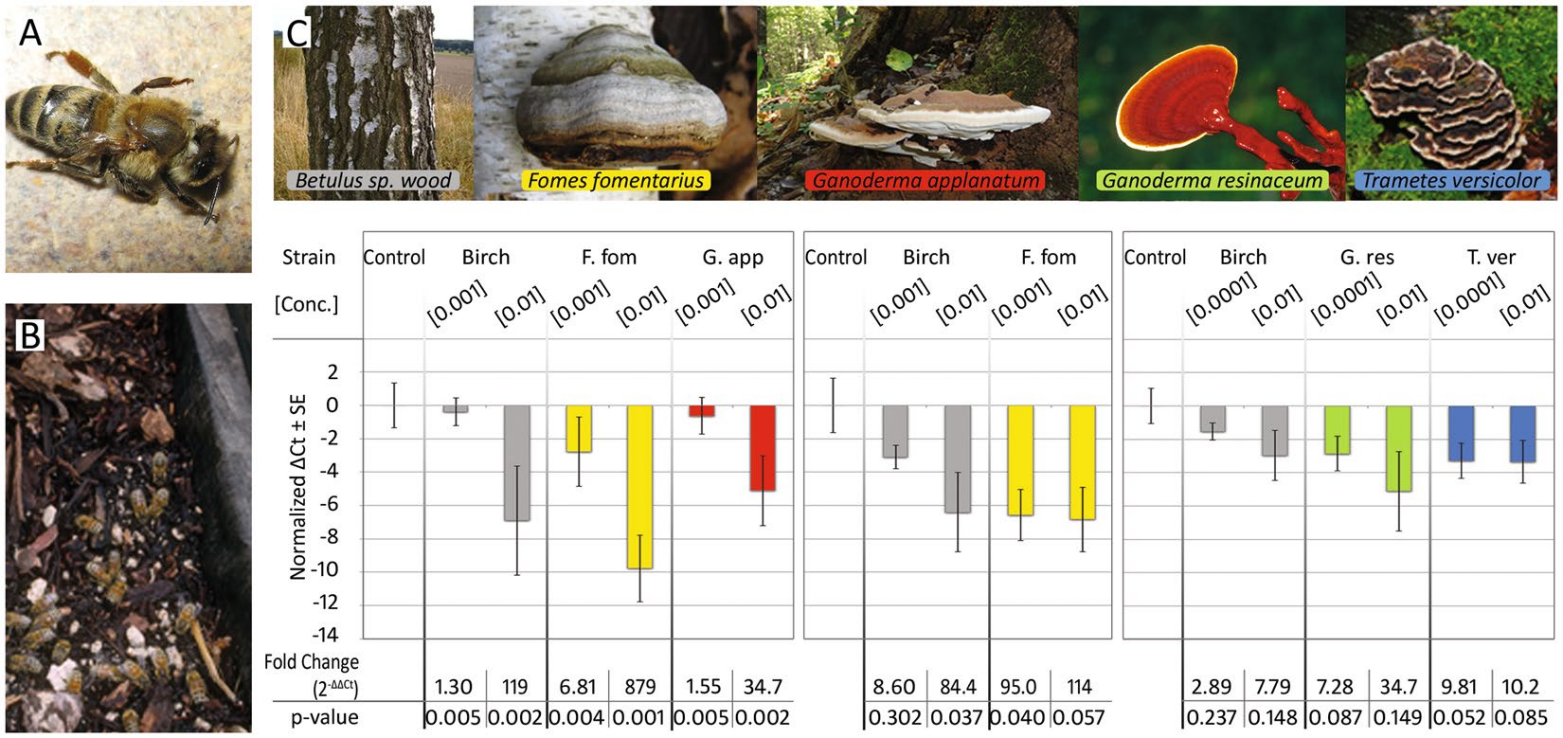
Extracts from fungi reduced deformed wing virus (DWV) in honey bees. (**A**) DWV shortens the lifespan of workers and causes developmental abnormalities in honey bees including deformed wings. (**B**) Honey bees forage on fungal mycelium where they ingest liquid exudates. (**C**) Bees fed extracts from polypore mycelium exhibited lower DWV virus titers. In three different trials, mixing fungal extracts into caged bees’ sucrose syrup food significantly reduced the levels of DWV as assayed by qPCR. Notably, the extracts of *F. fomentarius* mycelium reduced DWV more than 800-fold in the first trial (2-tailed t-test, n = 10, t = 1.12, p = 0.005). For each trial, qPCR ΔCt values are normalized to mean value of the respective control groups. Photos: Xolani90-https://commons.wikimedia.org/wiki/File:Deformed_Wing_Virus_in_worker_bee.JPG - [CC BY-SA 3.0 (https://creativecommons.org/licenses/by-sa/3.0)]-no changes (**A**), PES (**B**, *Fomes*, *G. resinaceum*), Titus Tscharntke – Wikimedia Commons (*Betula*), George Chernilevsky-Wikimedia Commons (*G. applanatum*), Norbert Nagel -https://commons.wikimedia.org/wiki/File:Trametes_versicolor_-_Coriolus_versicolor_-_Polyporus_versicolor_-_Schmetterlingstramete_-_Bunte_Tramete_-_Schmetterlingsporling_-_01.jpg [CC BY-SA 3.0 (https://creativecommons.org/licenses/by-sa/3.0)]-cropped (*Trametes*).

Two of the most important factors contributing to widespread colony losses are infestation of *A. mellifera* with the parasitic mite *Varroa destructor* and the suite of associated viruses^5–7^. The extent to which *Varroa* mites are involved in the amplification and dissemination of RNA viruses among honey bee populations has only recently become apparent, with *Varroa* infestation now known to be associated with at least 10 honey bee viruses^8–10^. Viruses are recognized to play a contributing role in widespread colony losses, especially deformed wing virus (DWV) and Varroa destructor virus-1 (VDV1)^6,7,11–13^. DWV is a devastating virus that causes shriveled wings, reduced worker life span, reduced foraging, and immunosuppression in honey bees^14,15^ (Fig. 1A). In addition to mite-mediated transmission, RNA viruses (including DWV) can also be transmitted among pollinators via pollen^16,17^. Another potentially problematic virus associated with honey bees and *Varroa* mites is the Lake Sinai virus group^9,18^. LSV was first identified in 2010^19^ but is now widespread in US honey bee colonies.

Currently, beekeepers are only able to indirectly control virus levels by using miticides to reduce mite infestation rates in managed honey bees. Overall, this effort has worked with only limited success, given the rapidity with which *Varroa* mites have developed resistance to synthetic miticides^20,21^. Another potential approach would be to reduce virus levels directly in honey bees by using a functional antiviral material, but no such products are currently available.

There is evidence that some fungi produce substances with demonstrable antiviral activity^22–25^. For example, alcohol or chloroform extracts from mycelial cultures and fruiting bodies of several polypore mushrooms (Order Polyporales) are known to have activity against pox virus, HIV-1 and H1N1 influenza^24–27^. Honey bees have been observed foraging directly on mycelium growing in outdoor beds^28^ (Fig. 1B), leading to speculation that they may be procuring a nutritional or medicinal gain. This behaviour may represent a novel facet of social immunity, given that a growing body of evidence indicates that honey bees self-medicate using plant-derived substances^29–31^. In this study, we evaluated extracts derived from the mycelia of several polypore mushroom species for activity against two major honey bee viruses *in vivo* in both laboratory and field studies. In both cases, reductions in DWV and LSV titers occurred in bees that were fed mycelial extracts in sucrose syrup.

## Results and Discussion

Laboratory experiments with caged bees showed that oral treatment with mycelial extracts from wood conk species significantly reduced the level of DWV. The effect of treatment with fungal extracts, regardless of species, was highly significant compared to caged bees fed only sugar syrup (DWV sugar controls vs. all mycelium treatments: 2-tailed t-test, n = 58; t = 4.33, p = 0.0001, ΔΔCt fold change = 23.0). *Fomes fomentarius* (amadou conk) and *Ganoderma applanatum* (artist’s conk) extracts exhibited significant antiviral activity in a dose-dependent manner (Fig. 1C). The strongest performing extract, a 1% (v/v) *F. fomentarius* extract:sugar syrup mix, reduced DWV over 800-fold in caged honey bees compared to the sugar syrup control (2-tailed t-test, n = 10, t = 1.12, p = 0.005, ΔΔCt fold change = 879).

**Figure 2:**
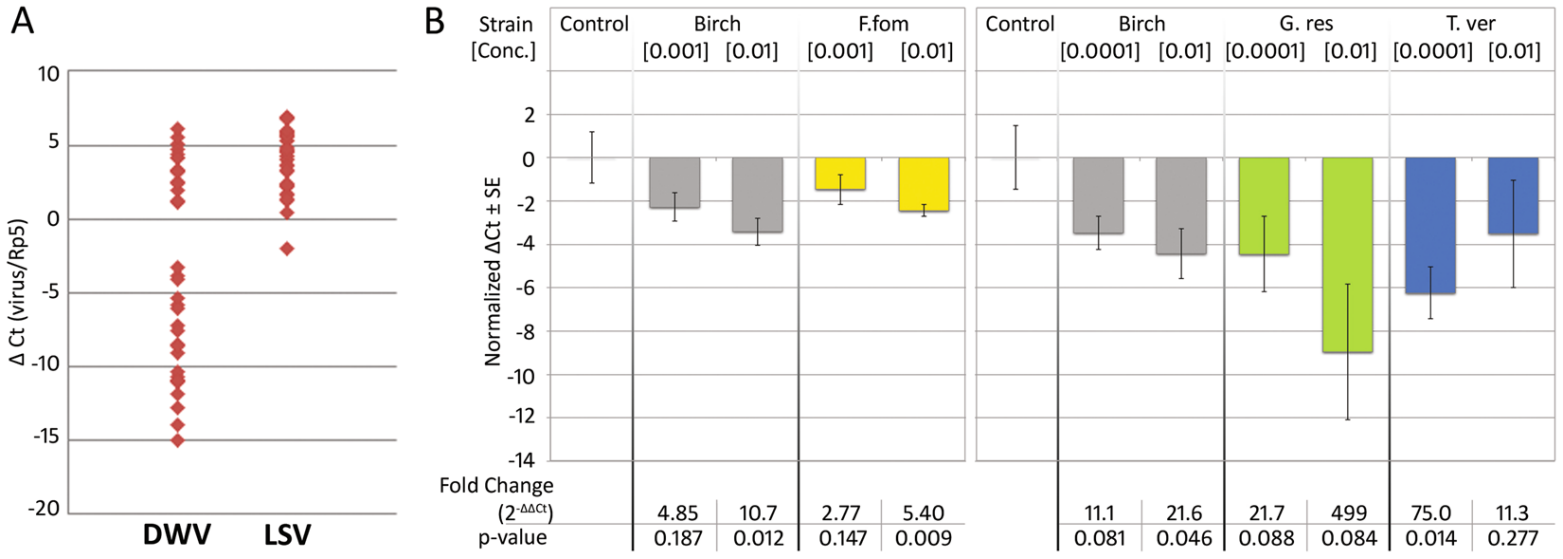
Lake Sinai virus (LSV) is reduced by fungal extract treatments. (**A**) All 36 individual bee abdomens from our source population tested positive for both DWV and LSV. LSV was more highly expressed than DWV, and more highly expressed than the honey bee *RpS5* reference gene. (**B**) Fungal extracts reduced LSV titers in bees after 7 days of treatment. *F. fomentarius* significantly reduced LSV at a 1% dosage (2-tailed t-test, n = 8, t = 3.96, p = 0.009, ΔΔCt fold change = 5.40). The greatest fold change difference was seen with *G. resinaceum* extract where treatment reduced LSV levels in bees 499-fold compared to bees fed only sucrose syrup (2-tailed t-test, n = 8, t = 2.59, p = 0.084).

Like DWV, LSV is also highly prevalent in honey bees in the Americas and Europe^9^. We tested 36 individual bee abdomens sourced from the initial population and found 100% of individuals tested positive for both DWV and LSV (Fig. 2A). LSV showed higher levels of virus load than DWV, and LSV was frequently more highly expressed than the reference gene used for qPCR (*Ribosomal protein 5*). Treating bees in cages with fungal extracts reduced the levels of LSV (Fig. 2B), with *F. fomentarius* (2-tailed t-test, n = 8, t = 3.96, p = 0.009, ΔΔCt fold change = 5.40) and *T. versicolo*r (2-tailed t-test, n = 8, t = 3.28, p = 0.0136, ΔΔCt fold change = 75) showing significant reductions. *Ganoderma resinaceum* demonstrated the greatest average reduction compared to control cages (2-tailed t-test, n = 8, t = 2.59, p = 0.084, ΔΔCt fold change = 499), but also the greatest variance among extracts tested. The overall effect of treatment with fungal extracts, regardless of species, was highly significant compared to caged bees fed only sugar syrup (LSV sugar controls vs. all mycelium treatments: 2-tailed t-test, n = 33, t = 3.98, p = 0.0004, ΔΔCt fold change = 21.1).

**Figure 3:**
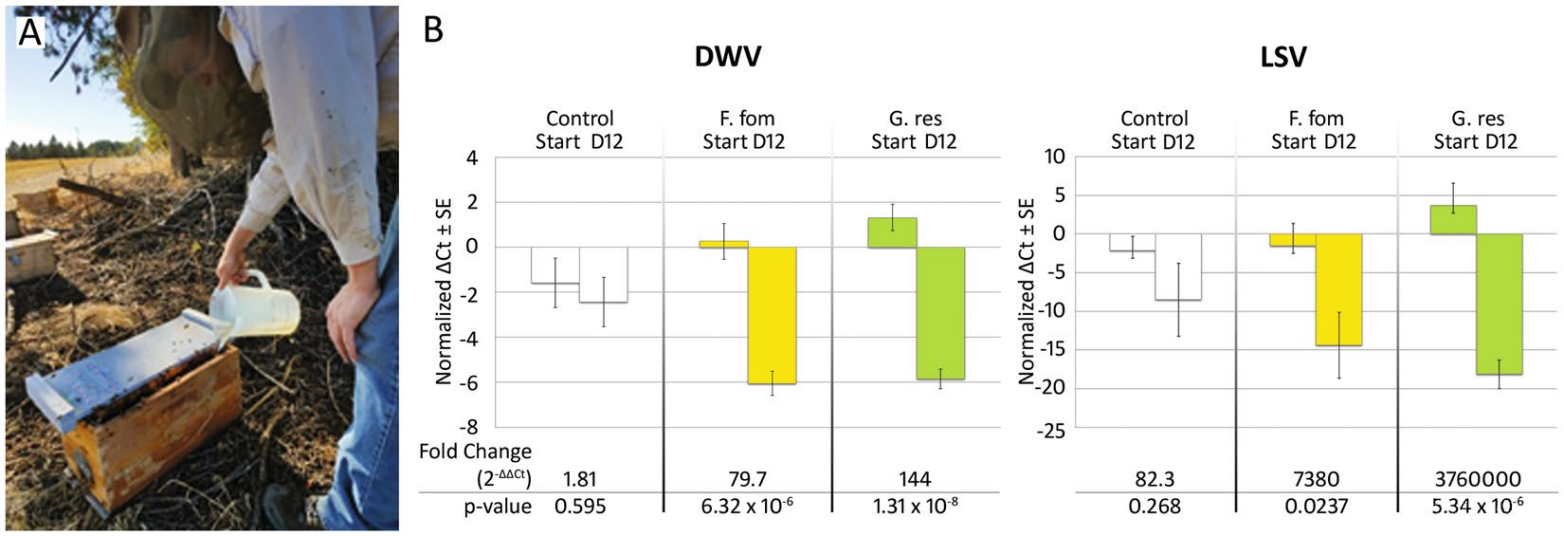
Fungal extracts reduce DWV and LSV levels in field trials. (**A**) Fungal extracts were mixed into sucrose solution and fed to bees using in-hive feeders commonly used in beekeeping. **(B)** Both *Fomes* and *Ganoderma* extracts significantly reduced DWV and LSV levels in field trials using 5-frame colonies with bees divided from a common population. Bees were sampled for virus levels at the start of treatment and 12 days later. Colonies fed *G. resinaceum* extracts exhibited a 79-fold greater reduction in DWV and a 45,000-fold greater reduction in LSV compared to controls fed sugar syrup. Quantitative PCR ΔCt values are normalized to the mean starting virus levels across all groups. Photo: WSS.

The two extracts exhibiting greatest fold reductions against DWV and LSV in the cage studies were selected for validation in a field trial. Small experimental colonies were treated once with 3 L of 1% fungal extract in 1:1 sucrose solution or sucrose solution only (Fig. 3A). Quantitative PCR analysis revealed that both DWV and LSV were reduced in treated colonies 12 days later (Fig. 3B). For DWV, hives treated with *F. fomentarius* extract showed a significant 79.7-fold reduction in viral levels (2-tailed t-test, n = 18, t = 6.58, p = 6.32 × 10^−6^, ΔΔCt fold change = 79.7), 44 times greater than in control colonies. *G. resinaceum* extracts also significantly decreased DWV levels (2-tailed t-test, n = 20, t = 9.75, p = 1.31 × 10^−8^, ΔΔCt fold change = 144) with treated colonies exhibiting 79.6-fold greater viral reductions than control colonies. The treatment effects from the fungal extracts were more pronounced with LSV. Although control colonies showed some reductions (2-tailed t-test, n = 18, t = 1.15, p = 0.267, ΔΔCt fold change = 82.3), *F. fomentarius* treatment lowered LSV levels 87.9 times greater than the control colonies (2-tailed t-test, n = 18, t = 2.50, p = 0.0238, ΔΔCt fold change = 7,380). The largest reduction in viral levels in these experiments were from *G. resinaceum* treatments where LSV levels were decreased 45,000 times greater than in control colonies (2-tailed t-test, n = 20, t = 6.37, p = 5.34 × 10^−6^, ΔΔCt fold change = 3.76 × 10^6^).

In addition to the demonstrated antiviral activity of the polypore mushroom mycelial extracts, extracts from non-inoculated fungal growth substrate (birch wood) also showed some activity against DWV and LSV. Even though the birch sawdust did not show visible signs of fungal infection (See Supplemental Fig. S1), many or most free-living forest trees have cryptic endophytic and saprophytic fungal associates^32,33^ and many of these symbiotic endophytes provide fitness advantages to the host^34,35^. To evaluate this possibility, samples of the birch sawdust from this study were analyzed using next generation sequencing, and multiple species of fungi were found to be present. Three common birch associated fungi accounted for 99.5% of all mapped reads: *Graphostroma platystoma*, *Chondrostereum purpureum*, and *Trametes versicolor*. This raises the possibility that saprophytic and endophytic fungi, co-extracted with the birch wood, may have contributed to the activity found in the extracts, including in the uninoculated birch wood. Further studies are needed to evaluate the contributing role of these fungi relative to any activity toward viruses caused by intrinsic birch phytochemicals such as betulinic acid^36,37^.

This study demonstrates that extracts of several polypore mushrooms reduced RNA virus titers in honey bees *in vivo*. Viruses, including the DWV and LSV groups, have been reported to play a significant role in the global pattern of declining honey bee health^7,9–13^, but no approved antiviral materials are currently available for beekeepers. Viruses typically associated with honey bees are widespread among non-*Apis* wild pollinators^38^, highlighting the importance of developing a means to control virus infections in managed populations. The mycelial  extracts tested here are orally active and readily consumed by bees, suggesting potential applications for beekeepers that provide critical pollination services. In addition to the potential direct impacts on honey bee health, the antiviral activity of fungal extracts can provide a research tool for the further exploration of the complex interactions between mites, viruses, and honey bee health.

## Methods

### Fungal mycelial extracts preparation

The following mycelial species/strains were collected and cultured by Paul Stamets:

*Fomes fomentarius,* Ithaca, New York

*Ganoderma applanatum*, Duckabush Valley, Washington

*Trametes versicolor*, Kamilche Point, Washington

The *Ganoderma resinaceum* culture originated from an anonymous source in Ontario, Canada.

Mycelial cultures were grown in sterile Petri dishes containing sterilized malt yeast agar (Fungi Perfecti, Shelton, WA). After three to four weeks of colonization in a clean room laboratory, the cultures were aseptically transferred into a 1000 mL Eberbach stirrer containing 800 mL of sterilized water. The mycelium was fragmented in the Eberbach container using a Waring blender base. The mycelial broth was then diluted 1:10 into sterilized water and transferred under sterile conditions into polypropylene incubation bags containing approximately 3 kg sterilized brown rice, which had been adjusted to approximately 45% moisture content prior to sterilization. An aliquot of 50–100 mL of diluted fluid was transferred into each of the 3 kg sterilized rice bags under sterile conditions. The fresh mycelial cultures were then incubated for 30–60 days, depending upon species, in a HEPA controlled clean room. Subsequently each mycofermented rice bag was distributed into 20 polypropylene bags of sterilized birch (*Betula papyrifera*) sawdust (2 kg each) and incubated for 30–60 days depending upon species.

Once colonization was determined to be sufficient (see Supplementary Fig. S1), the mycelium-colonized substrate was frozen to arrest growth, then transferred to HDPE containers for extraction. The myceliated substrate was mixed with a 50% ethanol/water solution (prepared by mixing equal weights of 95% organic ethyl alcohol and spring water) at twice the weight of the myceliated substrate, agitated, and then macerated at room temperature for 3–5 days. The mixture was pressure filtered and the supernatant decanted into containers for storage at 4 °C. The final product contains ethanol, fungal mycelial compounds and possibly unutilized growth substrate constituents. All extracts were prepared under a standardized manufacturing process and were used in subsequent tests as crude extracts of the solid substrate fermentation without further purification or characterization.

### Cage and field treatments with fungal extract

For the cage studies, a large population cage was filled with approximately 10 kg of worker honey bees pooled from multiple colonies residing in a single apiary in Pullman, Washington, USA. Each experimental cage (n = 5 per treatment) was populated with approximately 300 worker bees each from the population cage and maintained in the laboratory with *ad libitum* sources of water and 1:1 sucrose/water syrup w/v or mycelial extracts (1%, 0.1% or 0.01% v/v in 1:1 sucrose/water syrup). Samples of 50 bees were collected from each replicated cage/treatment on day 7 and frozen until viral titer analysis. Cages where 50% or more of individuals died over the course of the experiment were not used for subsequent analyses. Three trials were run and individual bees were sampled from the population cage in the second cage trial to establish the individual virus levels in Fig. 2A.

For the field studies, 30 five-frame “nucleus” colonies, each with a laying queen and approximately 8,000 worker bees were established near Moscow, Idaho in September 2016. To create colonies with equalized starting viral levels, approximately 30 kg of worker honey bees were collected from multiple colonies residing in Washington State University experimental apiaries (Pullman, Washington) and placed in a large screened population cage. To stock the nucleus colonies, approximately 1 kg of adult worker bees was removed from the pooled population cage and added to each experimental colony. Nucleus colonies were maintained outside and allowed free flight and normal foraging throughout the experiment. All colonies were sampled before treatment to determine a viral baseline level and again 12 days following treatment. Ten replicate colonies were each fed mycelial extracts of *F. fomentarius* or *G. resinaceum* at a concentration of 0.01 (1% extract) in 3 liters of 1:1 sucrose/water syrup (extracts) or fed 1:1 sucrose syrup only (control).

### Viruses - Sample processing, q-PCR, and statistical analyses

To analyze virus levels from a cage or nucleus colony, 50 mL of bees (~60–100 individuals) were collected onto dry ice and then stored at −80 °C. From each sample, 50 individual bees were then pooled for molecular analysis. Bees were homogenized in a disposable RNA extraction bag with 20 mL guanidine thiocyanate lysis (GITC) buffer, and nucleic acids were extracted using acid phenol protocol^39^. The nucleic acids were treated with DNase I at 37 °C for 1 h followed by 10 min at 75 °C. First-strand complementary DNA (cDNA) was generated from 2 μg total RNA using a master mix containing 50 U Superscript II (Invitrogen), random primer set (7-mer at 10- mM concentration), 2 nmol dNTP mix, 2 nmol polydT-18, and 0.1 nmol polydT (12–18). The cDNA synthesis was carried out at 42 °C for 50 min followed by 15 minutes at 70 °C^37^. Samples were screened for honey bee viruses via quantitative real-time PCR (1 ul cDNA template in a 20 ul reaction) using Bio-Rad SsoFast™ SYBR® Green Supermix, 96-well optical PCR plates, and a Bio-Rad CFX Connect™ thermal cycler. Positive controls (purified PCR product) and non-template controls (nuclease-free H2O) were included in each run. The thermocycler was programmed for enzyme activation at 95 °C for 30 seconds, followed by 50 cycles of denaturation at 95 °C for 5 seconds and annealing/extension 60 °C for 30 seconds.

Primer sequences used for qPCR are listed below. DWV and RpS5 primer sequences are from Englesdorp *et al*.^38^, and the sequence for LSV is from R. Cornman at the USDA-ARS (unpublished data). Both DWV and LSV primers are designed to target areas of genetic conservation in the viruses, allowing detection of the majority of genotypes.

*A.mel-*RpS5.F: AATTATTTGGTCGCTGGAATTG

*A.mel-*RpS5.R: TAACGTCCAGCAGAATGTGGTA

DWV.F: GAGATTGAAGCGCATGAACA

DWV.R: TGAATTCAGTGTCGCCCATA

LSVF: GTCATCCCAAGAGAACCACTYAC

LSVR: CRCACYGACATGAAGAAATGAGGTC

Viral levels were determined using the ∆Ct method^40^, normalizing the virus target expression to honey bee *RpS5* reference gene expression. Statistics and standard errors were calculated using these ∆Ct values (which are log_2_ based). Fold change was then calculated using the ∆∆Ct method^38^. For cage trials, ∆∆Ct was calculated by directly comparing treatment cages to control cages. For field trials where a baseline viral level was established, ∆∆Ct was calculated within each treatment or control group and comparisons were made by dividing treatment ∆∆Ct by control ∆∆Ct. For graphical representation in Figs 1 and 2, ∆Ct values were normalized to the average viral values for the control colonies within that trial. Samples for which the *RpS5* reference gene did not amplify by 40 Cts were removed. Because some hives cleared the virus to below qPCR detection levels, a value of Ct = 51 was assigned to any sample where the virus was not detected by the end of the qPCR run at Ct = 50. Student’s t-tests were performed using 2-tails and unequal variance in RStudio (RStudio Inc., Boston, MA, USA).

### Sequencing

Because endophytes are common in mature trees, we tested uninoculated birch sawdust for the presence of endogenous fungi using next generation sequencing. This sawdust was used to make the birch wood extracts and served as the final growth substrate for the wood decay fungi used to make the fungal extracts. Authentechnologies (Richmond, CA) used proprietary universal primers for fungi to amplify 11,460 reads sequenced using an Ion Torrent Personal Genome Machine Next Generation DNA Sequencer.

## Electronic supplementary material

Supplementary Information

## Data Availability

The datasets generated during and/or analysed during the current study are available from the corresponding author on reasonable request.
