# Supplementary material for: Extracts of Polypore Mushroom Mycelia Reduce Viruses in Honey Bees
Source: Sci Rep. 2018 Oct 4;8:13936. doi: 10.1038/s41598-018-32194-8 (PMC6172205; doi:10.1038/s41598-018-32194-8)
Supplement: Supplementary file 1 — Supplementary Information [file 41598_2018_32194_MOESM1_ESM.docx]

**Extracts of Polypore Mushroom Mycelia Reduce Viruses in Honey Bees**

Paul E. Stamets, Nicholas L. Naeger, Jay D. Evans, Jennifer O. Han, Brandon K. Hopkins, Dawn Lopez, Henry M. Moershel, Regan Nally, David Sumerlin, Alex W. Taylor, Lori M. Carris, Walter S. Sheppard

Supplementary Information

**Visual comparison of uninoculated and myceliated sawdust**

Figure 1.

The amount of fungal biomass present in the solid substrate fermentation was quite substantial and was macroscopically apparent as thick white mycelium growing throughout the birch media. In contrast, the uninoculated birch sawdust was a light brown with no visible mycelium and no binding together of sawdust particles as typically encountered in sawdust with actively growing fungi. Based on these significant qualitative differences between the birch substrate and the solid substrate fermentation, it is likely that the incidental amount of fungal DNA detected by next-gen sequencing represented an insignificant source of fungal metabolites compared with the quantities present in the inoculated treatments.


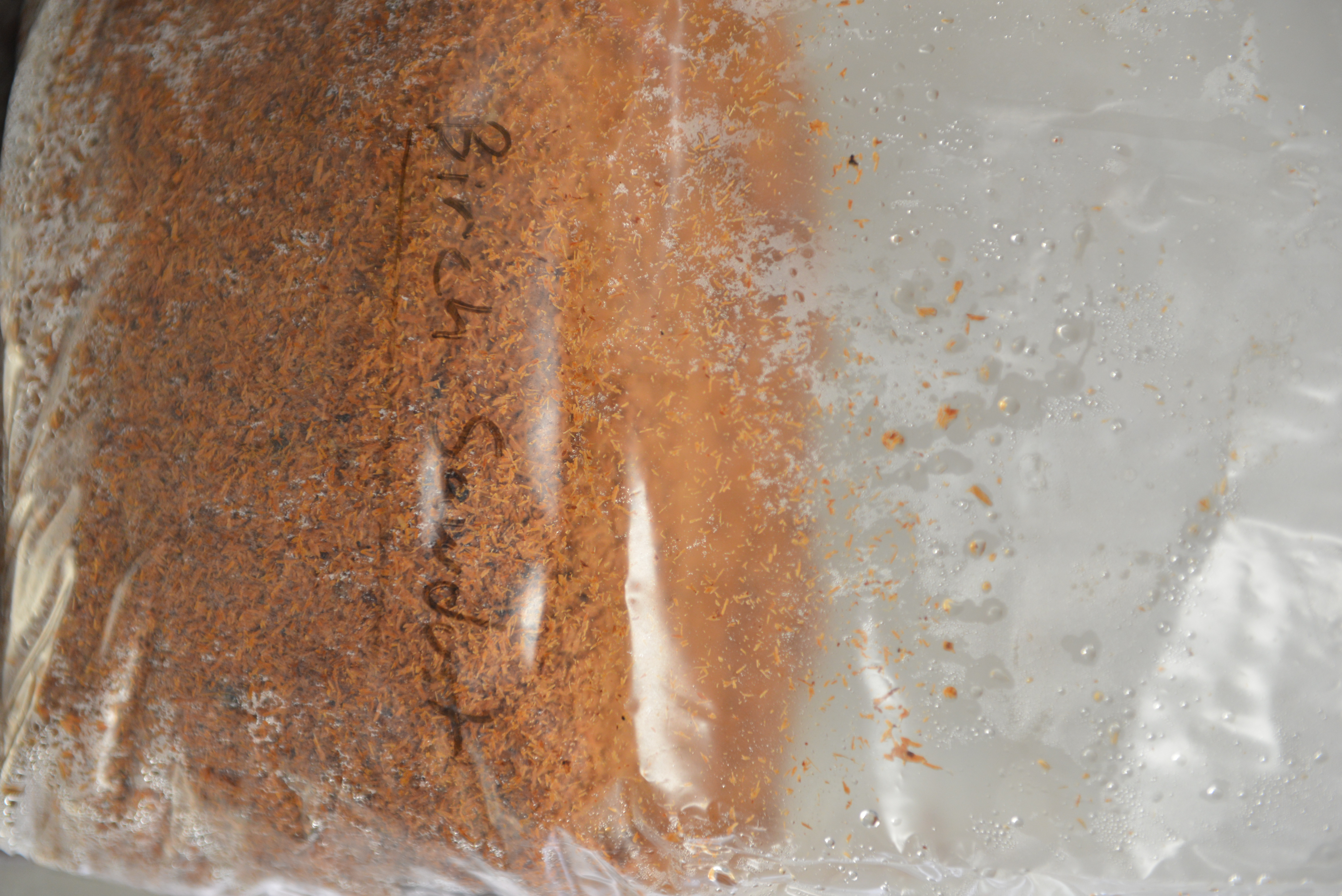

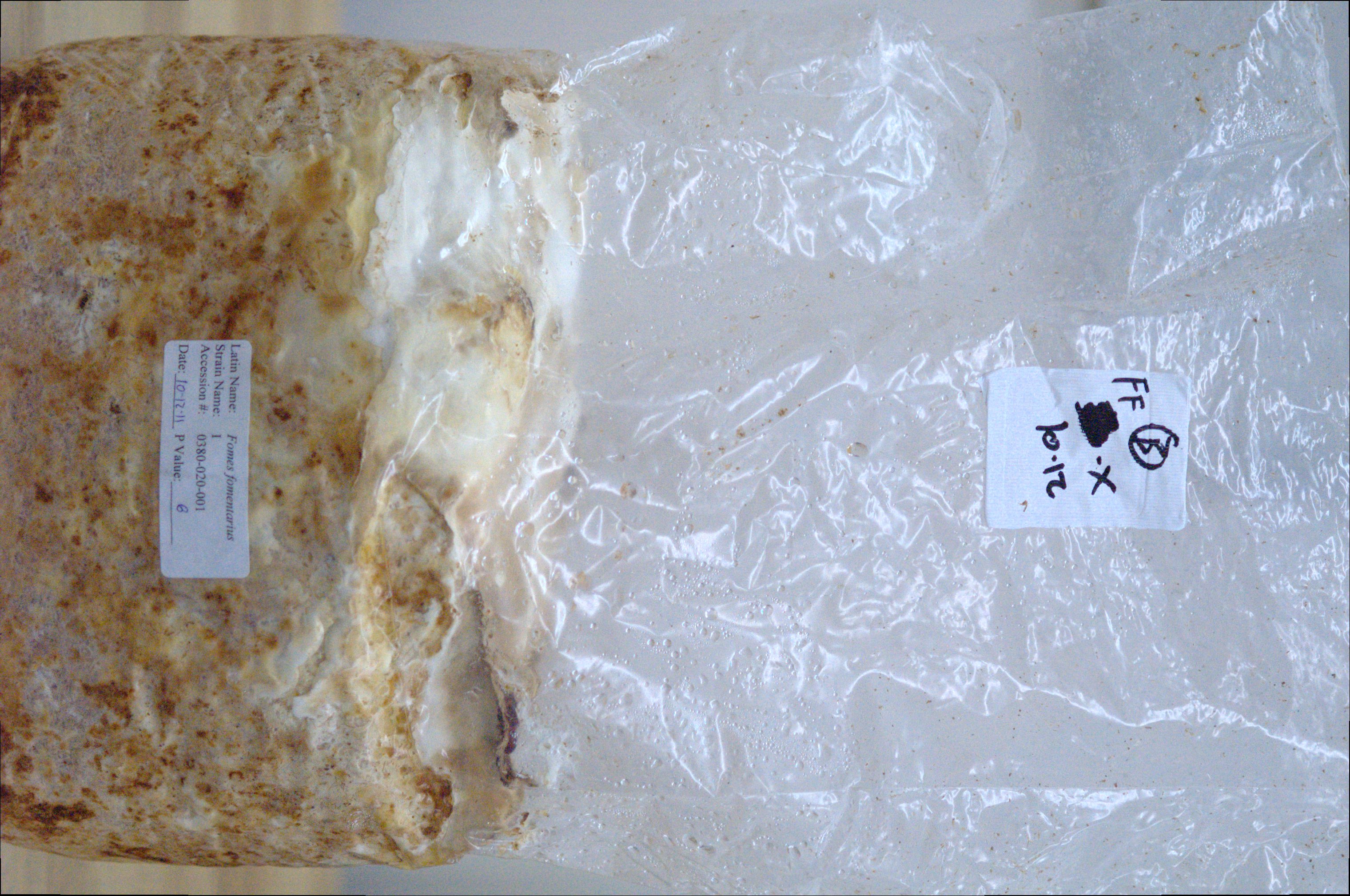


Caption: (left) sterilized birch sawdust substrate. (right) axenic solid substrate fermentation of sterilized birch substrate after incubation with *F. fomentarius*, just prior to extraction. The mottled white/brown in the figure on the right is thick laminated mycelium, and is representative of the degree of colonization of the other tested mushroom species.
